# Arthroscopic Intra-articular Biceps Tenodesis With All-Suture Anchor

**DOI:** 10.1177/26350254241301446

**Published:** 2025-02-27

**Authors:** Kevin C. Wang, Jeffrey J. Theismann, Elliot Cole, Alan L. Zhang

**Affiliations:** * Department of Orthopedic Surgery, University of California, San Francisco, San Francisco, California, USA; Investigation performed at University of California, San Francisco, San Francisco, California, USA

**Keywords:** biceps tenodesis, SLAP tear, biceps tendinitis, arthroscopy, rotator cuff tear

## Abstract

**Background:**

Biceps tenodesis can be used to treat both superior labral anterior and posterior (SLAP) tears and degenerative biceps pathologies in the setting of rotator cuff pathology or as stand-alone techniques. Research has shown that arthroscopic biceps tenodesis has comparable outcomes to open tenodesis with the benefit of being less invasive.

**Indications:**

Surgical indications for intra-articular tenodesis of the long head of the biceps (LHB) include SLAP tear, failed SLAP repair, partial-thickness LHB tear, biceps instability, tenosynovitis, and clinical examination consistent with LHB pain.

**Technique Description:**

We present our technique for arthroscopic intra-articular biceps tenodesis with a top-of-the-groove all-suture anchor using percutaneous suture passage with a spinal needle and polydioxanone suture. Sutures are shuttled through the LHB in situ before releasing the tendon from the labrum.

**Results:**

The authors have seen excellent clinical results using this method without any cases of tenodesis failure. Prior studies have shown subpectoral, suprapectoral, and top-of-the-groove biceps tenodesis to have similar good clinical outcomes at multiple time points.

**Discussion/Conclusion:**

This technique is advantageous as it is time efficient and cost-effective, and tension on the biceps can be easily maintained as suture is passed through the tendon in situ before the tendon is released from the superior labrum.

**Patient Consent Disclosure Statement:**

The author(s) attests that consent has been obtained from any patient(s) appearing in this publication. If the individual may be identifiable, the author(s) has included a statement of release or other written form of approval from the patient(s) with this submission for publication.

## Graphical abstract

**Graphical Abstract:**
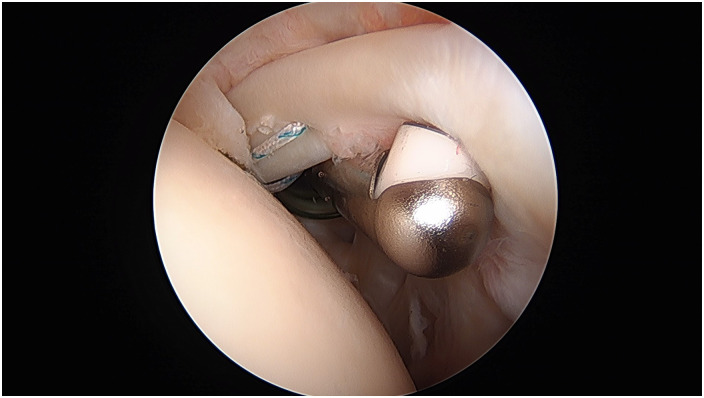
This is a visual representation of the abstract.

## Video Transcript

This is our surgical technique video for arthroscopic intra-articular biceps tenodesis with all-suture anchor. The authors are Kevin Wang, Jeff Theismann, and Elliot Cole from the University of California, San Francisco sports medicine and shoulder surgery fellowship and our faculty mentor, Dr. Alan Zhang.

These are our disclosures.

## Background

Biceps tenodesis can be used to treat both superior labral anterior and posterior (SLAP) tears or degenerative biceps pathologies in the setting of rotator cuff pathology or as stand-alone techniques. Research has shown that arthroscopic biceps tenodesis has comparable outcomes to open tenodesis with the benefit of being less invasive.

We present our technique for arthroscopic intra-articular biceps tenodesis with a top-of-the-groove all-suture anchor using percutaneous suture passage.

## Indications

Surgical indications for intra-articular tenodesis of the long head of the biceps (LHB) include SLAP tear, failed SLAP repair, partial-thickness LHB tear, biceps instability, tenosynovitis, and clinical examination consistent with LHB pain.

Contraindications include partial tearing or tenosynovitis distal to the planed intra-articular tenodesis level, complete/retracted LHB tear, and fixed/irreducible LHB dislocation.

Our patient is a 36-year-old man with left shoulder pain for 2 years. The pain is worse with lifting and pull-ups. Due to the pain, he has stopped going to the gym and playing the guitar. He has tried treatment in the form of physical therapy, nonsteroidal anti-inflammatory drugs, and 2 previous glenohumeral joint cortisone injections, which provided temporary relief.

On physical examination, the patient has full active range of motion, 5/5 strength of the rotator cuff in all planes, and positive Speed, Yergason, and O’Brien tests. His clinical examination is most consistent with SLAP tear as well as biceps irritation.

Multiple views on radiographs of the left shoulder show no fracture, dislocation, or narrowing of the glenohumeral joint. Magnetic resonance imaging (MRI) of the left shoulder shows no biceps pathology or tearing in the bicipital groove. Coronal MRI demonstrates a SLAP tear with a paralabral cyst. As a result of the imaging and clinical findings and age over 35 years, the patient was indicated for surgical treatment with arthroscopic biceps tenodesis.

For our procedure, the patient is placed in a beach-chair position per senior surgeon preference, but this procedure can be performed in the lateral decubitus position as well. We use a pneumatic arm holder to help with arm positioning.

In terms of equipment and implants, we use a standard 30° arthroscope, a double-loaded all-suture anchor, a 6-mm disposable cannula, an 18-gauge spinal needle, a 0-polydioxanone suture (PDS), an arthroscopic grasper with teeth, an arthroscopic loop grasper, an arthroscopic knot pusher, and a radiofrequency ablator as well as an arthroscopic shaver.

## Technique Description

Before the start of the surgery, the shoulder was injected with 1% Lidocaine with epinephrine to help with hemostasis. A standard posterior viewing portal is used. This is a video of a left shoulder in the beach-chair position. A diagnostic arthroscopy is performed, and an anterior working portal is established in the medial aspect of the rotator interval in line with the glenoid face. A disposable 6-mm × 72-mm cannula is inserted.

A probe is used to evaluate the superior labrum. This patient has findings of a type 2 SLAP tear as the superior labrum and biceps anchor are unstable and can be lifted off during probing. The decision to proceed with arthroscopic biceps tenodesis was made.

An all-suture anchor is then placed at the top of the bicipital groove. The insertion cannula for the all-suture anchor is placed percutaneously into the joint lateral to the anterior working portal in the rotator interval. In this case, we prefer a 2.3-mm all-suture anchor double loaded with a No. 2 high-tensile suture. A pilot hole for the suture is drilled through the cortical bone on the humeral head with a positive stop in the cannula. The all-suture anchor is then tapped into the bone through the same insertion cannula. The cannula is removed, and the all-suture anchor is set in place by pulling on all 4 limbs of the suture slowly at the same time.

Next, an 18-gauge spinal needle is used for suture shuttling. The needle is passed percutaneously through the skin, lateral to the insertion point of the anchor and directly through the LHB. The location of the needle entry into the tendon can be adjusted based on the desired tension on the tendon. A 0-PDS is passed through the spinal needle and retrieved with a grasper through the working cannula.

A loop grasper is then used to retrieve 1 limb of the No. 2 suture from the all-suture anchor through the working cannula (in this case, it is the striped suture). Outside of the working cannula, a simple knot is made to tie the PDS around the No. 2 suture from the anchor. The other end of the PDS on the lateral aspect of the shoulder is then used to pull the knotted end of the PDS back through the biceps and out of the joint, which effectively shuttles the No. 2 striped suture from the anchor through the biceps tendon.

Next, a loop grasper is used to retrieve the end of the No. 2 striped suture that was passed through the biceps out the working cannula. The suture is found on the anterior aspect of the biceps in the far lateral corner of the rotator interval. Not shown in this video, but the matching No. 2 striped suture that was not passed through the biceps can now be retrieved from the anchor by the loop grasper through the working cannula in preparation for future tying. This creates a simple suture configuration.

The process for suture shuttling through the tendon with the spinal needle is then repeated with the opposite No. 2 suture strand from the double-loaded all-suture anchor (in this case, the solid colored suture was passed next). This results in 2 simple sutures (1 striped and 1 solid colored) passed through the biceps tendon with the limbs through the tendon to be used as the post for arthroscopic knot tying. Before knot tying, the biceps tendon is released from the superior labrum with a radiofrequency ablator or arthroscopic scissors. The No. 2 sutures are then sequentially tied down to their matching limbs using alternating half-hitches in the working cannula.

## Results

Final evaluation of the biceps tenodesis shows no movement through shoulder and elbow range of motion.

## Discussion

Pearls and pitfalls for this technique are listed.

When setting up the case, it is important for your anterior working portal to be in the medial aspect of the rotator interval, approximately at the level of the face of the glenoid. This will allow for percutaneous placement of the all-suture anchor and suture passage lateral to the working portal. This will also ensure that the disposable cannula placed in the working portal does not block the percutaneous passage anchor or spinal needle for suture shuttling through the LHB.

Alternatively, if your working portal anteriorly is placed too laterally, it can make both placement of the anchor and passage of spinal needle difficult. If the percutaneous passage of the spinal needle through the biceps is too lateral, it can be difficult to retrieve the suture after it has been shuttled through the biceps. Finally, the proximal stump of the biceps can be debulked after tenotomy and before tying to help prevent mechanical symptoms or stump prominence.

Advantages of this technique include it being cost-effective with a PDS used to shuttle a stitch through the biceps instead of single-use suture-passing instruments. The LHB tension is easily maintained as the suture is passed through the tendon in situ before the tendon is released from the superior labrum, allowing the surgeon to set the tension. This technique can be used in the presence of subscapularis tearing or biceps instability. The all-suture anchor also minimizes the bony footprint on the humeral head as it is typically less than 2.5 mm in diameter. Finally, this technique is time efficient as it does not require a separate open incision or the need to localize the biceps extra-articularly after it has been tenotomized either open or arthroscopically. When the 2.3-mm all-suture anchor pulls out or there is severe LHB tendinopathy, an open subpectoral tenodesis can still be performed.

Disadvantages of this technique include that it cannot adequately address biceps pathology if it extends into the bicipital groove distal to the site of percutaneous suture passage. There are limited suture configurations when using a spinal needle to pass through the tendon as we mainly use either simple or mattress configurations, while with an open biceps tenodesis, a running locking Krakow stitch can be used. In this technique, we prefer to use simple configurations to minimize the risk of suture entanglement. Using a Mason-Allen–type suture may be a stronger configuration, but there can be greater risk for suture entanglement during passing.

## Conclusion

When analyzing research on patient outcomes for different bicipital tenodesis techniques, prior studies have assessed onlay versus inlay, intra-articular/suprapectoral versus subpectoral, and whether certain techniques may be better for patients with bicipital groove pain.^4^

Jackson et al^3^ performed a systematic review and meta-analysis in 2022 and reported no significant difference between onlay or inlay biceps tenodesis when comparing patient-reported outcomes (PRO) scores and slightly increased Popeye deformity with the inlay technique (11.28%) versus the onlay technique (7.8%). No difference in failure rates or cramping rates was found. Deng et al^1^ published a systematic review and meta-analysis in 2020 comparing open subpectoral with arthroscopic suprapectoral biceps tenodesis for SLAP tears or biceps abnormalities and showed no significant differences in Popeye deformity, residual pain, American Shoulder and Elbow Surgeons scores, or Constant scores.

Guerra et al^2^ published in a 2023 study with 1923 patients that subpectoral, suprapectoral, and top-of-the-groove biceps tenodesis had similar good clinical outcomes at multiple time points. Kelly et al^5^ in 2021 performed a cadaveric analysis to assess movement of the LHB tendon after suprapectoral intra-articular tenodesis and showed that there was less than 2 mm of motion in all arm positions, which may be why groove pain is improved with this technique despite the LHB not being removed from the bicipital groove.

These are our references.

Thank you for your time, and we hope this shows a technique that may benefit your surgical practice.
